# Significant correlation between urinary N^1^, N^12^-diacetylspermine and tumor invasiveness in patients with clinical stage IA non-small cell lung cancer

**DOI:** 10.1186/s12885-015-1068-5

**Published:** 2015-02-18

**Authors:** Yusuke Takahashi, Hirotoshi Horio, Koji Sakaguchi, Kyoko Hiramatsu, Masao Kawakita

**Affiliations:** 1 Department of Thoracic Surgery, Tokyo Metropolitan Cancer and Infectious Diseases Center Komagome Hospital, 3-18-22 Hon-komagome, Bunkyo-ku, Tokyo Japan; 2 Department of General Thoracic Surgery, Teikyo University School of Medicine, 2-11-1 Kaga, Itabashi-ku, Tokyo Japan; 3 Center for Medical Research Cooperation, Tokyo Metropolitan Institute of Medical Science, 2-1-6 Kami-kitazawa, Setagaya-ku, Tokyo Japan; 4 Department of Thoracic Surgery, Nagano Prefectural Suzaka Hospital, 1332 Oaza-suzaka, Suzaka, Nagano Japan

**Keywords:** Tumor invasiveness, Urine diacetylspermine, Clinical stage IA, Non-small cell lung cancer

## Abstract

**Background:**

To select optimal candidates for limited lung resection, it is necessary to accurately differentiate the non-invasive tumors from other small-sized lung cancer. Urinary N^1^, N^12^-diacetylspermine (DiAcSpm) has been reported to be a useful tumor marker for various cancers. We aimed to examine the correlation between preoperative urinary DiAcSpm levels and specific clinicopathological characteristics such as the histological tumor invasiveness in patients with clinical stage IA non-small cell lung cancer (NSCLC).

**Methods:**

We defined non-invasive tumors as NSCLC showing no vascular invasion, lymphatic permeation, pleural invasion, or lymph node metastasis. Preoperative urine samples were obtained from 516 consecutive patients with NSCLC resected at our institution between April 2008 and January 2013. Urinary DiAcSpm values were determined for all preoperative urine samples using the colloid gold aggregation procedure. Among these patients, 171 patients with clinical stage IA NSCLC met the criteria of our study cohort. Finally, we investigated the correlation between non-invasive tumor and urinary DiAcSpm levels.

**Results:**

The median urine DiAcSpm for males was 147.2 nmol/g creatinine and 161.8 nmol/g creatinine in females. These median values were set as the cut-off values for each gender. Patients with higher urinary DiAcSpm levels frequently had significantly elevated serum CEA (p = 0.023) and greater lymph node metastasis (p = 0.048), lymphatic permeation (p = 0.046), and vascular invasion (p = 0.010). Compared with patients with non-invasive tumors, patients with invasive tumors had a tumor size >2.0 cm (p = 0.001), serum CEA >5.0 mg/dL (p < 0.001), high urinary DiAcSpm (p = 0.002), and a tumor disappearance rate (TDR) <0.75 (p < 0.001). Multivariate analysis revealed that a tumor size < 2.0 cm (RR = 2.901, 95% CI; 1.372-6.136, p = 0.005), high urinary DiAcSpm (RR = 3.374, 95% CI; 1.547-7.361, p = 0.002), and TDR < 0.75 (RR = 4.673, 95% CI; 2.178-10.027, p < 0.001) were independent predictors for invasive tumors.

**Conclusions:**

We successfully showed that there was a significant correlation between urinary DiAcSpm levels and pathological tumor invasiveness in patients with clinical stage IA NSCLC. Further research would elucidate the clinical usefulness of DiAcSpm levels as a predictor of tumor invasiveness.

**Electronic supplementary material:**

The online version of this article (doi:10.1186/s12885-015-1068-5) contains supplementary material, which is available to authorized users.

## Background

Surgery is one of the major therapeutic choices for patients with primary lung cancer. Specifically in clinical stage IA non-small cell lung cancer (NSCLC), the standard treatment remains lobectomy and systematic hilar and mediastinal lymph node dissection [1]. Recent advancements in diagnostic techniques have increased the accuracy and frequency of detection of small-sized lung tumors [2]. Using these advancements, a number of researchers have attempted to prove the effectiveness of limited lung resection; however, their studies have shown a higher local recurrence rate after limited resection, even though a negative surgical margin had been confirmed pathologically [3-5]. Possible explanations for local recurrence following limited resection may include insufficient surgical margins, misdiagnosis of nodal involvement, or intrapulmonary lymphatic spread [6]. Limited resection is often performed in patients with peripheral small-sized lung cancer, although two randomized control trials comparing limited resection with standard lobectomy in patients with clinical T1aN0M0 NSCLC are currently taking place in Japan. In order to select optimal candidates for limited resection it is necessary to accurately differentiate between non-invasive tumors that have been confirmed histologically and other small-sized lung cancers. Several researchers aiming to better characterize these tumors have reported that a greater proportion of ground-glass opacity (GGO) was a significant predictor of non-invasive lung cancer [7,8]. Moreover, a recent report described that the presence of a micropapillary component was independently associated with an increased risk of recurrence in patients with stage I NSCLC treated with limited resection [9].

N^1^, N^12^-diacetylspermine (DiAcSpm) is a minor component of urinary polyamines comprising less than 0.5% of the total polyamines in healthy human urine [10]. It has been widely accepted that actively proliferating cells tend to excrete more polyamines as a result of the activation of intracellular polyamine metabolism and turnover. Furthermore, the presence of DiAcSpm is found to be increased in advanced stage cancers [11]. In recent years, we have reported that urinary DiAcSpm is often significantly elevated in patients with various cancers, including early stage disease [11]. Based on this finding, we aimed to examine the correlation between preoperative urinary DiAcSpm levels and clinicopathological characteristics such as the histological invasiveness of tumors in patients with clinical stage IA NSCLC.

## Methods

Urine samples were obtained before treatment from 516 consecutive patients who were diagnosed with operable NSCLC at Tokyo Metropolitan Cancer and Infectious Disease Center Komagome Hospital between April 2008 and January 2013. Among these patients, 171 consecutive patients with clinical stage IA NSCLC were consistent with our study cohort. We received prior approval to use patient urine samples from the ethical committees at Tokyo Metropolitan Cancer and Infectious Disease Center Komagome Hospital and Tokyo Metropolitan Institute of Medical Science. Informed consent was obtained from all patients and approved by the Institution Review Board.

### Determination of urinary DiAcSpm using the colloid gold aggregation procedure

Urine samples were supplemented with 3 mmol/L NaN_3_ and stored at −20°C, as previously described [12]. Urinary DiAcSpm was measured by the colloidal gold aggregation procedure using a JCM BM-6010 automatic biochemical analyzer (JEOL, Tokyo, Japan). The colloidal gold aggregation procedure relies on the binding specificity of bovine serum albumin (BSA)-acetylspermine conjugate, a DiAcSpm mimic, to colloidal gold-antibody complexes resulting in a stable red-purple solution. Addition of the BSA-acetylspermine conjugate into the solution induces a color change from red-purple to grey due to aggregation of the colloidal gold particles. DiAcSpm, a monovalent antigen that cannot cross-link multiple gold particles, competes with the BSA-acetylspermine conjugate for binding to the colloidal gold-antibody complexes. Therefore, when a urinary sample containing DiAcSpm is added to the colloidal system, DiAcSpm competitively binds to the colloidal gold-antibody complexes and suppresses color change. Thus, by using this competitive colloidal system, the concentration of DiAcSpm in a urine sample can be determined by measuring the color change of the solution. Auto DiAcSpm® (Alfresa Pharma Co., Osaka, Japan), a reagent that can be used in automated clinical analyzers, is commercially available. The concentration of DiAcSpm determined by the colloidal gold aggregation procedure have been shown to closely correspond with those determined by mass spectrometric analysis [13]. Urine creatinine levels were measured enzymatically using the NESCAUTO® VLII CRE reagent (Alfresa Pharma Co., Osaka, Japan) on a JCM BM-6010 automated biochemical analyzer (JEOL, Tokyo, Japan).

### Clinicopathological assessment

All clinicopathological data were retrieved from patient medical records. Preoperative evaluation included a physical examination, blood chemistry analysis, measurement of tumor markers, bronchoscopy, chest radiography, computed tomography (CT), brain MRI, and bone scintigraphy. Integrated positron emission tomography scan and CT scan (PET/CT) were also performed where appropriate. Clinical lymph node metastasis was defined as enlarged lymph nodes measuring > 1 cm on the short axis by CT scan and/or hypermetabolic lymph nodes on PET/CT scans. Histological confirmation of lymph node metastasis was made using endobronchial ultrasound-guided transbronchial needle aspiration of enlarged lymph nodes. All patients underwent a lobectomy or bilobectomy and systematic lymph node dissection for resection of the primary lesion.

All surgical specimens underwent thorough pathological examination. Each tumor was diagnosed according to the current histological classification of the World Health Organization [14] and was staged according to the tumor node metastasis classification of the International Union against Cancer, 7^th^ edition [15]. Vascular and pleural invasion and lymphatic permeation were evaluated using both hematoxylin and eosin (HE) section staining and Victoria Blue van Gieson (VvG) section staining.

Non-invasive tumor was defined as NSCLC showing no vascular invasion, pleural invasion, lymphatic permeation, or lymph node metastasis. The following clinicopathologic information was collected from patient medical records: age (categorized into two groups, ≤69 years and >69 years, according to the median age), gender, tumor size, preoperative serum CEA level (dichotomized at the normal upper limit of 5 mg/dL), pathological lymph node involvement, vascular invasion, pleural invasion, lymphatic permeation, histological type, and pathological stage.

Of the 171 NSCLC lesions, 140 adenocarcinomas were identified and classified according to the adenocarcinoma classification newly proposed by the International Association for the Study of Lung Cancer, American Thoracic Society, and the European Respiratory Society (IASLC/ATS/ERS) [16]. The adenocarcinomas were divided into three groups: (1) adenocarcinoma in situ (AIS), (2) minimally invasive adenocarcinoma (MIA), and (3) invasive adenocarcinoma (I-ADC). Then, we evaluated the correlation between histological invasiveness of the tumors, defined according to the above classification, and the specific clinicopathological factors described above.

### Measurement of tumor disappearance rate on chest CT

We calculated a tumor disappearance rate (TDR) using the following tumor dimension measurements on high-resolution chest CTs [17]: pDmax, which is the maximum dimension of a tumor on pulmonary window setting images; pDperp, the largest dimension perpendicular to the maximum axis on pulmonary window setting images; mDmax, the maximum dimension of a tumor on mediastinal window setting images; and mDperp, the largest dimension perpendicular to the maximum axis on mediastinal window setting images. Using these measurements, the TDR was then calculated using the following formula:

$$ \mathrm{T}\mathrm{D}\mathrm{R} = 1\ \hbox{--}\ \left(\mathrm{mDmax} \times \mathrm{mDperp}\right)\ /\ \left(\mathrm{pDmax} \times \mathrm{pDperp}\right) $$

The TDR threshold was set at 0.75, as previously described [18].

### Statistical analysis

Two-category comparisons were performed using the Pearson Chi-Square (*χ*^2^) Test and the Fisher’s Exact Test for categorical variables, and the Mann–Whitney *U* Test was performed for continuous variables. All statistical tests were two-sided, and *p* < 0.05 were considered statistically significant. To determine the impact of factors considered significant predictors of survival by earlier univariate analysis, a multivariate analysis was performed on these predictors using a logistic regression model. All statistical analyses were performed using SPSS software (version 20; SPSS Inc., Chicago, III).

## Results

### Patient characteristics

Our cohort consisted of 84 males and 87 females. Ages ranged from 36 to 89 years, with a median age of 69 years. Tumor size from the resected specimens ranged from 0.8 to 3.5 cm, with a median of 1.8 cm. Within the study cohort, there were 137 adenocarcinomas, 24 squamous cell carcinomas, and 10 with other histology (2 adenosquamous carcinomas, 2 large cell carcinomas, 2 non-small cell carcinomas, 2 large cell neuroendocrine carcinomas, 1 clear cell carcinoma, and 1 carcinoid). There were 136 identified as pathological stage IA tumors, 121 as stage IB, 7 as stage IIA, and 7 as stage IIIA. Tumor stage was increased in 35 patients due to additional diagnoses following surgery. Discoveries that contributed to the up-staging included lymph node metastasis in 15 patients, an actual tumor size > 3 cm in 8 patients, and pleural invasion in 26 patients. Further, lymphatic permeation was seen in 24 patients and vascular invasion in 39 patients. A summary of patients and their pathological characteristics is presented in Table 1. The median urine DiAcSpm was 147.2 nmol/g creatinine (range: 3.7-3918.8) in males and 161.8 nmol/g creatinine (range: 66.0-867.6) in females. These median values were set as the cut-off values for urine DiAcSpm for each gender, as previous reports have demonstrated that the urine DiAcSpm values for healthy women are higher than those for men [12].

**Table 1:** **Baseline characteristics of initial study cohort (n = 171)**

Age	
Median (range)	69 (36–89)
**Gender**	
male	84
female	87
**Smoking history**	
Never-smoker	58
Smoker	113
**Synchronous multiple lung cancer**	
yes	4
no	167
**Histological type**	
adenocarcinoma	137
squamous cell carcinoma	24
large cell carcinoma	2
large cell neuroendocrine carcinoma	2
adenosquamous carcinoma	2
carcinoid	1
clear cell carcinoma	1
NSCLC, NOS	2
**Lymphatic permeation**	
negative	147
positive	24
**Vascular invasion**	
negative	132
positive	39
**Pleural invasion**	
negative	145
positive	26
**Pathological stage**	
IA	136
IB	21
IIA	7
IIB	0
IIIA	7

### Measurement of tumor disappearance rate on chest computed tomography

When we applied 0.75 as the cut-off value for TDR where TDR was used to test the correlation with pathological invasiveness, we achieved sensitivity and specificity values of 59.2% and 74.6%, respectively, with the best predictive accuracy of 70.2%. For urinary DiAcSpm, we applied cut-off values of 147.2 for males and 161.8 for females and achieved 69.4% sensitivity and 57.4% specificity which yielded a prediction accuracy of 60.8% for pathological invasiveness. When the TDR and urinary DiAcSPM were inversely combined, the predictive value of non-invasive tumor was 94.9% (37 of 39).

### Correlation between clinicopathological characteristics and urinary DiAcSpm

We evaluated the data to determine whether there was a correlation between urinary DiAcSpm and clinicopathological characteristics (Table 2). The high urinary DiAcSpm group often showed significantly elevated serum CEA (p = 0.023), lymph node metastasis (p = 0.048), lymphatic permeation (p = 0.046), and vascular invasion (p = 0.010) compared with the low urinary DiAcSpm group.

**Table 2:** **Correlation between urine DiAcSpm and clinicopathological factors**

Factors	Urine DiAcSpm level (nmol/g creatinine)		*p-v*alue *
	Low	High	
**Age (years)**			
≤69	47	40	
>69	38	46	0.286
**Smoking history**			
Never-smoker	32	26	
Smoker	53	60	0.335
**Tumor size (cm)**			
≤2.0	55	49	
>2.0	30	37	0.348
**Serum CEA level (mg/dL)**			
≤5.0	79	69	
>5.0	6	17	0.023
**TDR**			
≥0.75	25	24	
<0.75	60	62	0.867
**Histological type**			
adenocarcinoma	68	69	
non-adenocarcinoma	17	17	0.970
**Lymph node metastasis**			
N0	82	75	
N1-2	3	11	0.048
**Lymphatic permeation**			
negative	78	69	
positive	7	17	0.046
**Vascular invasion**			
negative	73	59	
positive	12	27	0.010
**Pleural invasion**			
pl0	75	70	
pl1-2	10	16	0.287

*Fisher’s exact test, DiAcSpm = diacetylspermine, CEA = carcinoembryonic antigen level.

The Mann–Whitney *U* test was used to evaluate differences between the absolute values of urinary DiAcSpm and serum CEA level (≤5.0 mg/dL vs. >5.0 mg/dL), lymph node metastasis (N0 vs. N1-2), vascular invasion (positive vs. negative), lymphatic permeation (positive vs. negative), histological type (adenocarcinoma vs. others), tumor size (≤2.0 cm vs. >2.0 cm), and TDR (≥0.75 vs. <0.75). The results of these tests are presented in Table 2 and are as follows: The urinary DiAcSpm from the normal serum CEA group and N0 group was significantly lower than the elevated serum CEA group (p = 0.044) and N1-2 group (p = 0.014), respectively. Urinary DiAcSpm from the negative vascular invasion group was significantly lower than the positive vascular invasion group (p = 0.002). Urinary DiAcSpm from the negative lymphatic permeation group was significantly lower than the positive lymphatic permeation group (p = 0.038). In contrast, there were no significant differences in urinary DiAcSpm in groups with adenocarcinoma (p = 0.585), tumor size ≤2.0 cm and >2.0 cm (p = 0.249) or TDR (≥0.75 and <0.75 (p = 0.489).

### Correlation between pathologically confirmed tumor invasiveness and clinicopathological factors

We investigated the relationship between pathologically confirmed tumor invasiveness and clinicopathological factors (Table 3). There were 122 cases of non-invasive tumors and 49 cases of invasive tumors. Tumor size >2.0 cm (p = 0.001), serum CEA >5.0 mg/dL (p < 0.001), high urinary DiAcSpm (p = 0.002), and TDR > 0.75 (p < 0.001) were more frequently observed in patients with invasive tumors than in those with non-invasive tumors. Pathologically confirmed tumor invasiveness was not significantly affected by age, gender, smoking history, or histological type.

**Table 3:** **Relationship between pathologic invasive factors and clinicopathological factors**

Factors	Invasive tumor	Non-invasive tumor	*p-v*alue*
**Age (years)**			
≤69	19	68	
>69	30	54	0.062
**Gender**			
male	30	54	
female	19	68	0.062
**Smoking history**			
Never-smoker	11	47	
Smoker	38	75	0.051
**Tumor size (cm)**			
≤2.0	20	84	
>2.0	29	38	0.001
**Serum CEA level (mg/dL)**			
≤5.0	35	113	
>5.0	14	9	<0.001
**Histological type**			
adenocarcinoma	35	102	
non-adenocarcinoma	14	20	0.090
**Urine DiAcSpm level (nmol/g creatinine)**			
low	15	70	
high	34	520	0.002
**TDR**			
≥0.75	4	45	
<0.75	45	77	<0.001

*Fisher’s exact test, DiAcSpm = diacetylspermine, CEA = carcinoembryonic antigen level, TDR = tumor disappearance rate.

### Multivariate analysis

We performed a multivariate analysis to determine independent predictors of pathologically confirmed invasive tumors (Table 4). A tumor size >2.0 cm (Risk ratio (RR) = 2.871, 95% confidence interval (CI): 1.347-6.119, p = 0.006), high urinary DiAcSpm (RR = 3.374, 95% CI; 1.547-7.361, p = 0.002), and TDR < 0.75 (RR = 6.103, 95% CI; 1.962-18.981, p < 0.001) were independent predictors of invasive tumors. However, serum CEA was not an independent predictor of pathologically confirmed tumor invasive tumors.

**Table 4:** **Multivariate analysis for prediction of non-invasive NSCLC among clinical stage IA patients**

Variables	Risk factors	Risk ratio for invasive tumor	95% CI	*p*-value*
**Tumor size (cm)**	>2.0	2.871	1.347-6.119	0.006
**Urine DiAcSpm level (nmol/g creatinine)**	high	3.374	2.736-7.361	0.002
**Serum CEA (mg/dL)**	≥5.0	2.316	0.841-6.377	0.104
**TDR**	< 0.75	6.103	1.962-18.98	<0.001

*Logistic regression analysis, CI = confidence interval, DiAcSpm = diacetylspermine, CEA = serum carcinoembryonic antigen level, TDR = tumor disappearance rate.

### Correlation between histological invasiveness defined in IASLC/ATS/ERS classification and clinicopathological characteristics

Of 171 NSCLC lesions, 140 stage IA adenocarcinomas were separately analyzed to determine the correlation between histological invasiveness, as defined by IASLC/ATS/ERS classification, and clinicopathological factors. We confirmed 87 cases of non-invasive adenocarcinomas and 53 cases of invasive adenocarcinomas. Male gender (p = 0.010), smoker (p = 0.033), tumor size >2.0 cm (p < 0.001), serum CEA >5.0 mg/dL (p = 0.006), high urinary DiAcSpm (p < 0.001), and TDR > 0.75 (p = 0.023) were more frequently associated with patients with invasive tumors than in patients with non-invasive tumors (Additional file 1: Table S1). Further, a tumor size >2.0 cm (RR = 3.249, 95% CI; 1.380-7.650, p = 0.007), high urinary DiAcSpm (RR = 8.208, 95% CI; 3.470-19.417, p < 0.001), and TDR < 0.75 (RR = 2.783, 95% CI; 1.090-7.108, p = 0.032) were independent predictors of invasive tumors (Additional file 1: Table S2). However, gender, smoking history, and serum CEA were not found to be significant independent predictors of histological invasiveness in clinical stage IA adenocarcinomas.

## Discussion

The extent of pulmonary resections for small-sized NSCLC remains a considerable concern for thoracic surgeons. The major challenge is determining which subgroups of NSCLCs are suitable candidates for limited resection. Several investigators have reported that pathologically confirmed invasive factors was not a rare occurrence and tumors showed a considerable recurrence rate, even when a patient’s tumor was classified as clinical stage IA NSCLC [1,19]. Currently, there are randomized controlled trials underway to compare the differences in outcome between a lobectomy and a limited resection in patients with clinical stage IA NSCLC.

Previous reports have shown that pathologically confirmed invasive factors from resected NSCLCs were strongly correlated with more frequent nodal involvement and poorer outcomes [19,20]. Based on these findings, it is widely accepted that the ability to predict pathologically non-invasiveness is essential for identifying optimal candidates for limited resection. A number of retrospective studies have indicated that tumor non-invasiveness was frequently confirmed in resected lung cancers smaller than 2 cm [8,20]. However, there are reports indicating the occurrence of lymph node metastasis in 6% to 12% of small-sized NSCLC [19]. In the current study, positive lymph node metastasis was observed in 14 of 171 (8.2%) patients, which is consistent with previous reports. According to these results, it is suggested that tumor size alone cannot predict the pathologic invasiveness of NSCLC.

Several reports have demonstrated that the TDR on High-Resolution Computed Tomography (HRCT) was a significant predictor of pathologically confirmed invasiveness in small-sized NSCLC [8,19]. Alternatively, it has been reported that the percentage of solid opacity of a tumor on HRCT is a useful predictor of a non-invasive tumor [20-22]. These reports also demonstrated that the TDR alone is insufficient to perfectly predict non-invasive tumors.

In this study we have successfully demonstrated that urinary DiAcSpm is a useful marker that significantly correlates with pathologically confirmed non-invasive tumors; thus, it may be used to identify suitable candidates for limited resection. DiAcSpm is one of the minor polyamine components secreted in human urine. Polyamine excretion increases with tumor cell proliferation via the activation of intracellular polyamine metabolism and turnover [23]. Chen and colleagues demonstrated that an increase in DiAcSpm levels is associated with the stimulation of oxidative catabolism of polyamines [24]. Moreover, Kuwata et al. recently reported that DiAcSpm levels were elevated in tumor tissues from both primary sites and liver metastasis, suggesting that DiAcSpm may be produced from cancer cells themselves [25].

We have previously reported that DiAcSpm is frequently elevated in patients with various cancers, including colorectal, breast, lung, prostate, testicular, renal, and pelvic cancer, with very low false-negative incidence [11,26]. It should be noted that compared to conventional tumor markers like CEA and CA19-9, urinary DiAcSpm level is more frequently elevated in the earlier stages of colorectal and breast cancer. In addition, it has been reported that poor prognosis of patients with urogenital malignancies is associated with an increase in urinary DiAcSpm [27]. Hiramatsu et al. also reported that there was a strong positive correlation between urinary DiAcSpm and disease progression [27], which is consistent with the current study results that high urinary DiAcSpm is positively correlated with tumor invasiveness (i.e., lymphatic permeation, vascular invasion, and lymph node metastasis). Therefore, we believe the results of the current study clearly indicate that urinary DiAcSpm level is significantly associated with pathologically confirmed tumor invasiveness in stage IA NSCLC.

The value for urinary DiAcSpm is usually normalized to creatinine (nmol DiAcSpm/g creatinine). Because DiAcSpm is not reabsorbed by the renal brush border, the glomerular clearance of DiAcSpm is comparable to that of creatinine [28]. This helps to explain why DiAcSpm, which is produced in tissues of early stage cancers and subsequently excreted into circulation, is recovered in the urine without significant loss and, therefore, may serve as a useful tumor marker that is highly sensitive for early stage cancers [11]. In one of our previous studies, we reported that the value for urinary DiAcSpm in a healthy male significantly differed from that in a healthy female [12]. We therefore separately employed the cut-off values for urinary DiAcSpm by gender.

We considered using TDR as a complement to urinary DiAcSpm due to its high specificity for predicting non-invasive tumors. TDR was confirmed to be independently correlated with pathological tumor invasiveness. Thus, we have shown that the combination of TDR and urinary DiAcSpm was strongly correlated with pathological invasiveness. Our further analysis evaluating the correlation between clinicopathological characteristics and histological invasiveness, as defined in the IASLC/ATS/ERS classification of adenocarcinomas (Additional file 1: Tables S1 and S2), strongly supported the results from the analysis of the original patient cohort (Tables 2, 3, 4). In fact, the latter analysis revealed a stronger correlation between histological invasiveness and urinary DiAcSpm.

The mechanisms underlying an increase in urinary DiAcSpm value in cancer patients have not been fully understood, although a considerable amount of literature has reported the usefulness of urinary DiAcSpm as a novel tumor marker. The current study and further investigation may contribute in clarifying the mechanism and clinical significance of DiAcSpm.

There are several limitations that may exist in the current study. First, the study involved retrospective data collection in a small cohort of patients, and there is a possibility for bias due to selecting clinical stage IA. Further, although the HRCTs were retrospectively reviewed by two experienced observers who were blind to patient identification, image evaluation by the observers is subjective, thus, our evaluations may lack reproducibility. Secondly, we could not definitively confirm the appropriate use of limited resection, because our data did not include survival data. Therefore, in a future study, we should perform a follow-up with patients in the current cohort.

## Conclusion

In conclusion, our data show that there is a significant correlation between urinary DiAcSpm and pathological tumor invasiveness in patients with clinical stage IA NSCLC. Future investigations should aim to elucidate the oncological significance and clinical usefulness of DiAcSpm.

## Additional file

Additional file 1: Table S1. Relationship between histological invasiveness in IASLC/ATS/ERS classification and clinicopathological factors in adenocarcinoma cases. **Table S2.** Multivariate analysis for prediction of invasive adenocarcinoma among clinical Stage IA patients.

## Abbreviations

NSCLC: Non-small cell lung cancer
GGO: Ground-glass opacity
DiAcSpm: N^1^, N^12^-diacetylspermine
BSA: Bovine serum albumin
CT: Computed tomography
PET: Positron Emission Tomography scan
HE: Hematoxylin and eosin
VvG: Victoria blue van Gieson
CEA: Carcinoembryonic antigen
TDR: Tumor disappearance rate
CI: Confidence intervals
HR: Hazard ratio
IASLC/ATS/ERS: The international association for the study of lung cancer, american thoracic society, and european respiratory society
AIS: Adenocarcinoma in situ
MIA: Minimally invasive adenocarcinoma
I-ADC: Invasive adenocarcinoma
