# Supplementary material for: Significant correlation between urinary N1, N12-diacetylspermine and tumor invasiveness in patients with clinical stage IA non-small cell lung cancer
Source: BMC Cancer. 2015 Feb 18;15:65. doi: 10.1186/s12885-015-1068-5 (PMC4391126; doi:10.1186/s12885-015-1068-5)
Supplement: Additional file 1: Table S1. — Relationship between histological invasiveness in IASLC/ATS/ERS classification and clinicopathological factors in adenocarcinoma cases. Table S2. Multivariate analysis for prediction of invasive adenocarcinoma among clinical Stage IA patients. [file 12885_2015_1068_MOESM1_ESM.doc]

Additional files

Table S1 Relationship between histological invasiveness in IASLC/ATS/ERS classification and clinicopathological factors in adenocarcinoma cases

| **Factors** | **AIS or MIA** | **Invasive adenocarcinoma** | ***p-v*alue** * |
| --- | --- | --- | --- |
| **Age (years)**  ≤69  >69 | 52  35 | 26  27 | 0.225 |
| **Gender**  male  female | 37  50 | 23  30 | 0.010 |
| **Smoking history**  Never-smoker  Smoker | 41  46 | 15  38 | 0.033 |
| **Tumor size (cm)**  ≤2.0  >2.0 | 65  22 | 26  27 | <0.001 |
| **Serum CEA level (mg/dL)**  ≤5.0  >5.0 | 82  5 | 42  　 12 | 0.006 |
| **Urine DiAcSpm level**  **(nmol/g creatinine)**  Low  High | 59  28 | 11  42 | <0.001 |
| **TDR**  　 ≥0.75  <0.75 | 33  54 | 10  43 | 0.023 |

*: Fisher’s exact test, DiAcSpm = diacetylspermine, CEA = carcinoembryonic antigen level; TDR: tumor disappearance rate; AIS: adenocarcinoma in situ; MIA: minimally invasive adenocarcinoma.

Table S2 Multivariate analysis for prediction of invasive adenocarcinoma among clinical Stage IA patients

| **Variables** | **Risk factors** | **Risk ratio for invasive tumor** | **95% CI** | ***p*-value*** |
| --- | --- | --- | --- | --- |
| **Gender** | male | 1.843 | 0.683-4.970 | 0.227 |
| **Smoking history** | smoker | 2.174 | 0.878-5.405 | 0.093 |
| **Tumor size (cm)** | >2.0 | 3.249 | 1.380-7.650 | 0.007 |
| **Urine DiAcSpm level (nmol/g creatinine)** | high | 8.208 | 3.470-19.42 | <0.001 |
| **Serum CEA (mg/dL)** | ≥5.0 | 1.143 | 0.328-3.978 | 0.834 |
| **TDR** | <0.75 | 2.783 | 1.090-7.108 | 0.032 |

*: Logistic regression analysis, CI = confidence interval, DiAcSpm = diacetylspermine, CEA = serum carcinoembryonic antigen level, TDR = tumor disappearance rate.
